# Both prokaryotes and eukaryotes produce an immune response against plasmids with 5ʹ-GTTTGTT-3ʹ

**DOI:** 10.1186/s13578-022-00825-3

**Published:** 2022-06-11

**Authors:** Nan Li, Dongya Jiang, Luqingqing He, Yunyun Yue, Qinxin Zhang, Shuang Wang, Yunfeng Zhang, Yuxuan Wei, Qingshun Zhao

**Affiliations:** 1 Model Animal Research Center, Medical School, Nanjing University, NanjingJiangsu, 210061 China; 2 School of Traditional Chinese Pharmacy, China Pharmaceutical University, NanjingJiangsu, 210009 China; 3 Department of Prenatal Diagnosis, Obstetrics and Gynecology Hospital, Affiliated to Nanjing Medical University, Nanjing Maternity and Child Health Care Hospital, Nanjing, 210004 Jiangsu China

**Keywords:** Eukaryotic cell, Prokaryotic cell, Innate immune memory, Defense response, Foreign plasmid, Core sequence, 5ʹ-GTTTGTT-3ʹ, ISG15, Transformation efficiency

## Abstract

In the evolutionary “arms race” from prokaryotes to eukaryotes, some memories of foreign DNA have been conserved for defensive purposes. Shortly after invasion by the plasmid, pEGFP-N1, the conserved the defense gene, *isg15*, was activated in the zebrafish zygote and in mammalian cells. Based on the sequence similarity, we found three virus-derived sequences in pEGFP-N1 which share the 5ʹ-GTTTGTT-3ʹ core sequence, an epigenetic factor leading to increased expression of *isg15*. Mutation of the core sequence greatly reduces the degradation rate of the plasmid in *E. coli* cells or zebrafish embryos. We conclude that a conserved defense response, common to both eukaryotic and prokaryotic cells, allows identification and degradation of plasmids containing 5ʹ-GTTTGTT-3ʹ.

**Supplementary Information:**

The online version contains supplementary material available at 10.1186/s13578-022-00825-3.

Bacteria and archaea have evolved at least three different intracellular immune strategies to combat phage infection: restricted modification (RM), CRISPR and the prokaryotic Argonaute (pAgo) system [1]. The RM system is an innate immune system designed to recognize modified foreign DNA sequences and is present in over 90% of sequenced bacterial and archaeal genomes [2]. Eukaryotic innate immunity consists of a broad defense system, producing a rapid inflammatory response to most pathogens or to tissue damage [3]. Moreover, free DNA in the cytosol activates the antiviral immune response mediated by interferons. In the current study, we pose the question of whether a lasting "immune memory" is retained by host cells.

## Injection of plasmid pEGFP-N1 caused abnormally increased expression of immune response-related genes in zebrafish zygotes

The zebrafish, *Danio rerio,* is a model animal widely used in biomedical research. The innate immune system can be detected at the zygote stage, while the adaptive immune system matures morphologically and functionally only 4–6 weeks after fertilization.

Transgenic zebrafish lines are often created by injection of the transgene construct in the form of a circular plasmid at the zygote stage. We aimed to investigate whether transcription of zygotic DNA would be changed in response to invasion by exogenous nucleic acids.

**Fig. 1:**
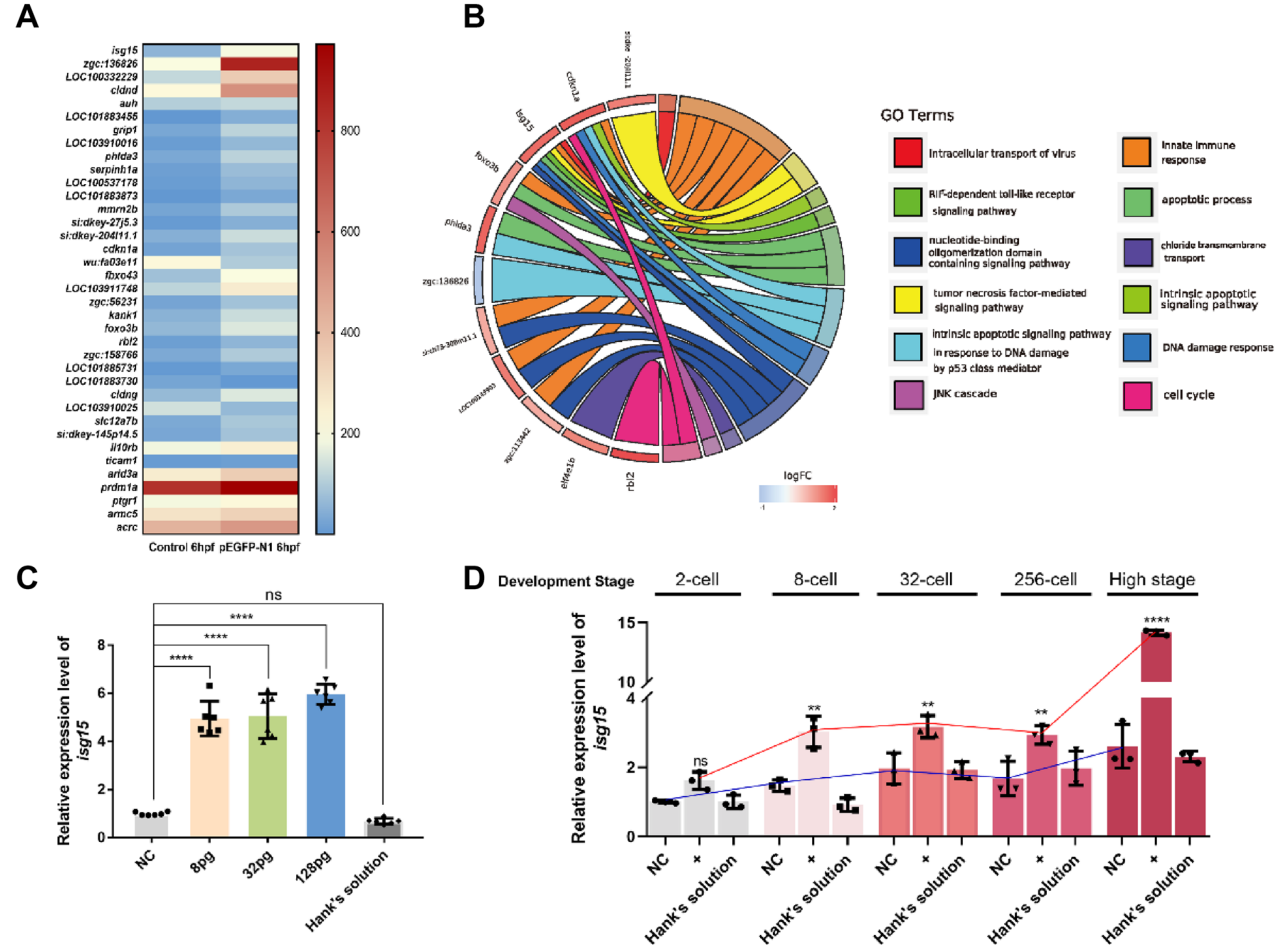
Expression of the zygotic gene, *isg15*, in zebrafish embryos after invasion by the plasmid, pEGFP-N1. **A** Heatmap to show normalized expression count differences of immune related genes from transcriptome sequencing at 6 hpf after injection of the foreign plasmid, pEGFP-N1, into 1-cell stage embryos. **B** Expression levels of six genes related to the apoptosis pathway, endogenous immune response and tumor necrosis factor-mediated pathway are up-regulated. **C** Up-regulation of *isg15* expression in 6 hpf zebrafish embryos by qRT-PCR. **D** Expression of *isg15* in 8-cell stage and High stage embryos after plasmid injection compared with controls. 8 pg, 32 pg, 128 pg: the injected amount of plasmid pEGFP-N1. NC: un-injected zebrafish embryos

Microinjection of the plasmid, pEGFP-N1, into zebrafish zygotes was followed by transcriptome sequencing. RNA samples were collected from embryos at intervals of 1 h-post-fertilization (hpf), 6 hpf and 12 hpf. Bioinformatics analysis showed that the expression of genes related to the endogenous immune response and tumor necrosis factor-mediated signaling pathways, including *isg15*, *foxo3b*, *phlda3*, *cdkn1a*, *zgc:136826* and *si:dkey-204l11.1,* were up-regulated (Fig. 1A, B) compared with controls which were not injected at 6 hpf (Additional file 1: Fig. S1A–J). Moreover, expression of *isg15* was significantly increased at the 8-cell stage compared with the non-injected group (Fig. 1D). These results demonstrate that the zygotic defensive gene, *isg15,* is activated in response to plasmid invasion and that the response was most obvious at 3 hpf (Fig. 1C; Additional file 1: Fig. S1A).

*isg15* is an IFN-stimulated gene involved in the response to infection by viruses and/or microorganisms [4]. Type I interferon is a lymphokine with extensive properties of immune regulation and is mainly secreted by innate immune cells, especially macrophages. However, it is generally considered that zebrafish only begin to display primitive macrophages at 22 hpf [5]. In order to confirm whether the increased expression of *isg15* is related to interferon, we tested the expression levle of IFN-related genes. Zebrafish has four IFN-related genes, *ifnphi1* is the ortholog gene of mammalian type I interferon. Interestingly, the expression of *ifnphi1*, *ifnphi4*, *cgasa*, *sting1* and *tnfa* was significantly lower in pEGFP-N1 injected group than in uninjected and Hank’s solution injected embryos. (Additional file 1: Fig. S1K, N-Q). And the expression level of *ifnphi2*, *ifnphi3* was no significant differences among these groups (Additional file 1: Fig. S1L-M). Therefore, the up-regulation of *isg15* may not be caused by type I interferon induction.

## The core sequence, 5ʹ-GTTTGTT-3ʹ, is key to up-regulation of *isg15* on invasion of eukaryotic cells by foreign DNA

Knockout of target genes using CRISPR/Cas9 technology causes the “genetic compensation effect” when sequence similarity is present. Some target mRNAs contain premature stop codons (PTC) after indel mutations and these sequences can be used as epigenetic modifiers to change the H3K4me3 level of adaptive genes, leading to increased transcription [6].

**Fig. 2:**
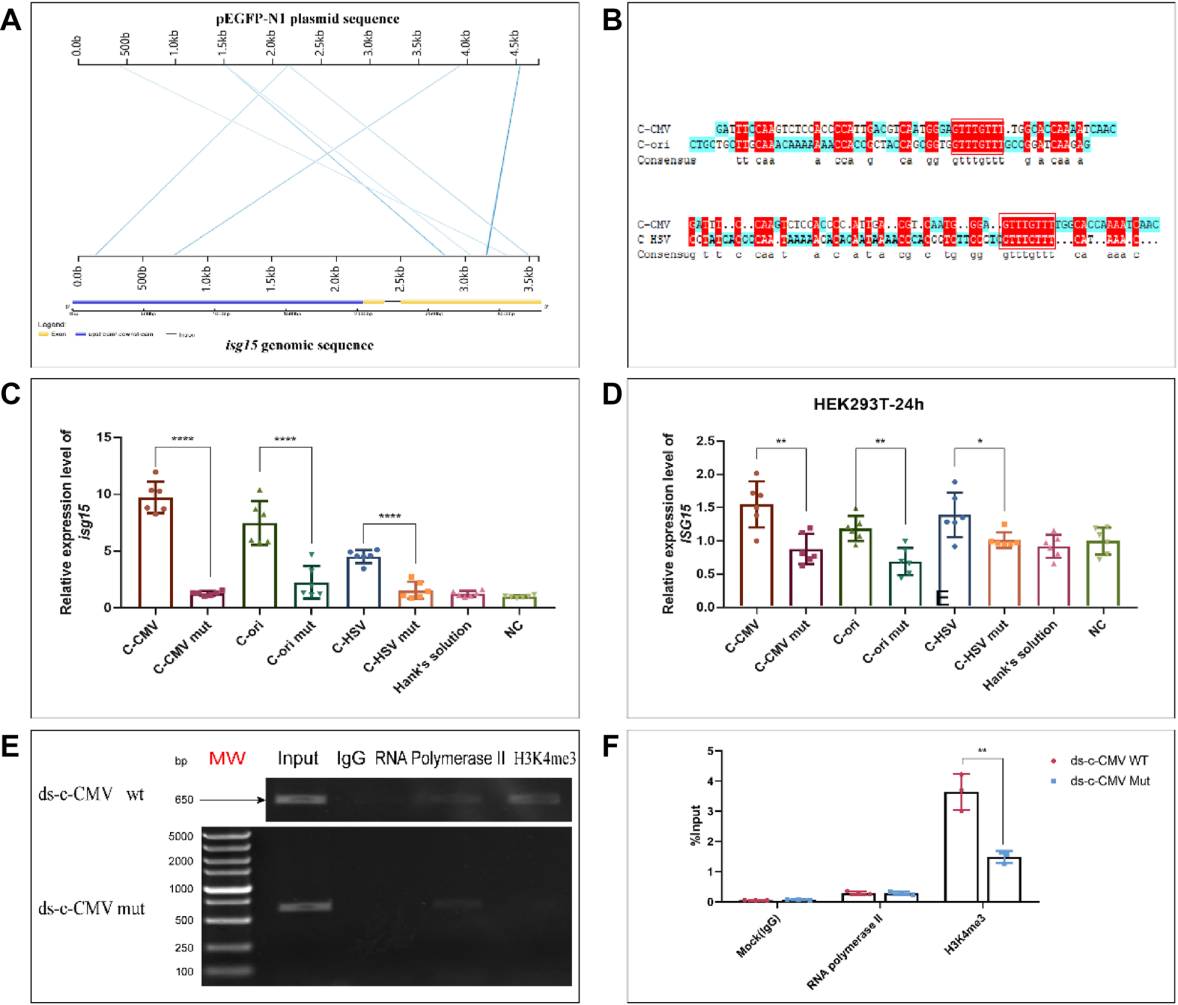
Plasmid pEGFP-N1 has core sequences regulating the expression of *isg15*. **A** BLASTN alignment of pEGFP-N1 plasmid and *isg15* gene sequences (including 2 kbp promoter upstream of TSS) with Kablammo (http://kablammo.wasmuthlab.org/). **B** Comparison of c-ori, c-CMV and c-HSV (DNAMAN) sequences showing a common 7 bp element, 5ʹ-GTTTGTT-3ʹ. **C** qRT-PCR results showing that the three mutated fragments of c-ori, c-CMV and c-HSV have weakened activation of *isg15* expression in 3 hpf embryos compared with the unmutated fragments. **D** Responses of HEK 293T cells to transfection with the three DNA fragments and their corresponding mutated sequences. Cells’ responses were significantly weakened after the three sequence mutations. **E**, **F** Semi-quantitative PCR and qRT-PCR results showing that the methylation level of H3K4me3 in the *isg15* promoter region was significantly increased. MW: DL5000 DNA Molecular Weight Marker

The plasmid, pEGFP-N1, consists of two sections: the plasmid backbone and EGFP encoding sequence (Additional file 1: Fig. S2A). By injecting linear and circular forms of the plasmid sequence into zebrafish embryos, we have established that the up-regulation of *isg15* is not related to plasmid structure (Additional file 1: Fig. S2B). BLASTN sequence comparison of the plasmid backbone and the entire gene sequence of *isg15,* including the 2 kbp promoter region, revealed three sequences within the pEGFP-N1 plasmid with sequence similarity to *isg15* by Kablammo (http://kablammo.wasmuthlab.org/) (Fig. 2A). These are the plasmid origin of replication, c-ori, present in most bacterial genomes; the CMV promoter, c-CMV, which derives from the herpes virus gene group and the HSV TK poly(A) signal, c-HSV, also from the herpes virus genome (Additional file 1: Fig. S2C). Sequences are shown in Additional file 1: Tables S1, S2.

As prokaryotic cells have evolved to form eukaryotic cells, phages and viruses have co-evolved to maintain the capacity for self-replication, generating an evolving host–pathogen relationship which we liken to an evolutionary “arms race” [7]. Therefore, we speculate that anti-viral immune memories might still be present in the early developmental stages of eukaryotic cells. The delivery of plasmids or virus-derived nucleic acids to zebrafish fertilized eggs or mammalian cells thus may activate the immune memory. Three fragments of DNA containing a 7 bp element, 5ʹ-GTTTGTT-3ʹ, were injected into fertilized zebrafish eggs and then the experiment repeated after mutation of the core sequence to 5ʹ-CAAACAA-3ʹ (Fig. 2B). The results showed that the three fragments containing the sequence, 5ʹ-GTTTGTT-3ʹ, could activate the expression of *isg15* but the mutant core sequence fragment, 5ʹ-CAAACAA-3ʹ, had lost the ability to up-regulate *isg15* (Fig. 2C). The same results were obtained when the experiments were repeated with a human embryonic kidney cell line (HEK 293T) and a human colon cancer cell line (HCT116) (Fig. 2D; Additional file 1: Fig. S2D). Thus, we conclude that the core DNA sequence, 5ʹ-GTTTGTT-3ʹ, plays an important role in stimulating the cell’s defense response.

What’s more, the expression level of other four genes have different response when c-CMV, c-HSV and c-ori injected into zebrafish embryos. Specifically, the expression level of *phlda3* was significantly activated when c-CMV and c-ori invaded, but not responded to c-HSV (Additional file 1: Fig. S3A). The expression level of *zgc:136826* and *foxo3b* was significantly activated when c-HSV invaded, but not responded to c-CMV and c-ori (Additional file 1: Fig. S3B, D). The expression level of *si:dkey-204l11.1* was significantly activated when c-ori invaded, but not responded to c-CMV and c-HSV (Additional file 1: Fig. S3C). These data show that the four genes have no response to all three DNA fragments with core sequence specifically.

In order to investigate whether an epigenetic mechanism was involved, a 61 bp double-stranded c-CMV fragment (ds c-CMV) and a c-CMV fragment with a mutant core sequence were injected into 1-cell stage zebrafish embryos for chromatin immunoprecipitation (ChIP) Assay. The results show a significantly higher level of H3K4me3 in the c-CMV-injected group compared with the mutant-injected group (Fig. 2E, F). Thus, DNA fragments may have an epigenetic effect and alter histone methylation levels [8]*.*

## The foreign plasmid core sequence activates expression of *isg15*

**Fig. 3:**
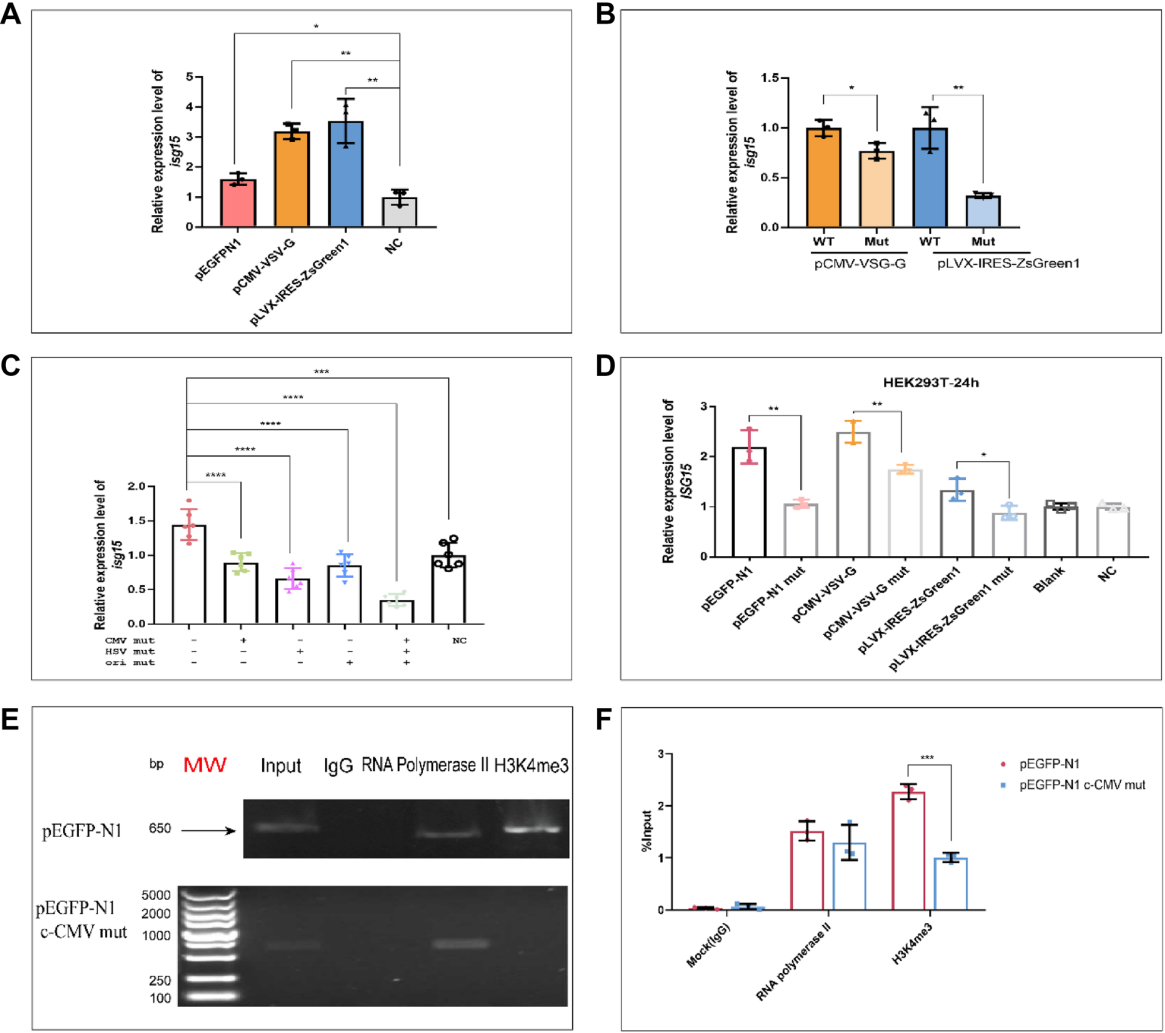
The pEGFP-N1 backbone has sequences that can regulate the expression of *isg15*. **A** qRT-PCR results showing that pEGFP-N1, pCMV-VSV-G and pLVX-IRES-ZsGreen1 activate the expression of *isg15* in 3 hpf embryos. **B** Mutation of core plasmid sequences reduces *isg15* expression. **C** qRT-PCR results showing that mutation of core sequences of ori, CMV and HSV in pEGFP-N1 reduces activation of *isg15* expression. **D** Mutation of the core sequence of pCMV-VSV-G and pLVX-IRES-ZsGreen1 weakens responses of HEK 293 T cells. **E**, **F** Semi-quantitative PCR and qRT-PCR results showing that the methylation level of H3K4me3 in the *isg15* promoter region was significantly increased. MW: DL5000 DNA Molecular Weight Marker

The role of the core sequence in the eukaryotic host defense response was further investigated by individually mutating the three core sequences in the CMV promoter, HSV poly(A) and ori of pEGFP-N1 to 5ʹ-CAAACAA-3ʹ (c-CMV mut, c-HSV mut, c-ori mut). A plasmid with all three sites simultaneously mutated was also generated (Amut) (Additional file 1: Fig. S4A). Each plasmid was injected into a 1-cell stage zebrafish zygote at the molar concentrations used previously. We observed that the response to foreign plasmids is significantly reduced when each individual core sequence is mutated and the reduction is more marked with the use of the three-sequence mutant, Amut (Fig. 3C). In addition, both the packaging vector plasmid, pCMV-VSV-G, and pLVX-IRES-ZsGreen1, which encodes the lentivirus and MuLV retrovirus envelope protein (Additional file 1: Fig. S4B, C), cause up-regulated expression of *isg15*, consistent with the results using pEGFP-N1 (Fig. 3A). When the core sequences were mutated, the response to the two virus-related plasmids was also significantly reduced (Fig. 3B). These results demonstrate that plasmids containing bacterial or viral sequences can stimulate an immune response in zebrafish zygotes.

And the core sequences on pEGFP-N1 were mutated and injected into zebrafish embryos, the expression of *phlda3*, *zgc:136826*, *si:dkey-204l11.1* and *foxo3b* were reduced. These data indicated that the core sequence on plasmid plays a vital role in the response to foreign plasmids for eukaryotic cells (Additional file 1: Fig. S3E–H).

The transfection experiments using unmutated plasmids and plasmids with one or more core sequences mutated were repeated using the mammalian cells, HEK 293T and HCT116. Expression of *isg15* was significantly reduced when the core sequence was mutated (Fig. 3D).

Based on the results described above, we put forward the hypothesis that invading DNA can stimulate an intracellular immune response directly without the involvement of type I IFN which is not available due to the lack of macrophage differentiation. Experiments in which the plasmid sequence was modified have established that the core sequence, 5ʹ-GTTTGTT-3ʹ, is vital in enabling expression of *isg15* in response to pathogenic DNA. We believe that this response may constitute the immune memory for invading nucleic acid formed during the evolution of eukaryotic cells. 

## Foreign plasmid containing the core sequence up-regulates *isg15* by enhancing the level of H3K4me3

The plasmids used in our experiments contain similar sequences to those in the *isg15* sequence, including the 2 kbp promoter, and such sequences play an important role in the up-regulation of *isg15.*

The ChIP assays were repeated following the injection of unmutated pEGFP-N1 or pEGFP-N1 with a core sequence mutation in c-CMV into 1-cell stage zebrafish embryos. Levels of H3K4me3 in the promoter of *isg15* were significantly lower following injection of the mutated sequence (Fig. 3E, F). Western blots also showed that after 24 h transfection of HEK 293T cells with either the wild-type plasmid or the c-CMV mutant plasmid, levels of H3K4me3 were significantly lower for the mutated sequence (Additional file 1: Fig. S2E). However, we also tested the H3K4me3 levels of other four genes, *foxo3b*, *zgc:136826*, *phlda3* and *si:dkey-204l11.1*. The results of semi-quantitative PCR showed that there were no significant changes in the H3K4me3 methylation levels in the promoter regions of the other four genes (Additional file 1: Fig. S4A–D). Combined with the results of Additional file 1: Fig. S3, it indicates that there are other reasons for pEGFP-N1 to activate the expression of these four genes. Through the alignment, we found that these four genes still have other consensus sequences with pEGFP-N1, and these sequences may play an important role in it.

## Macrophages autonomously phagocytose and degrade the invading foreign plasmids, which activates the defense response in eukaryotic organisms

**Fig. 4:**
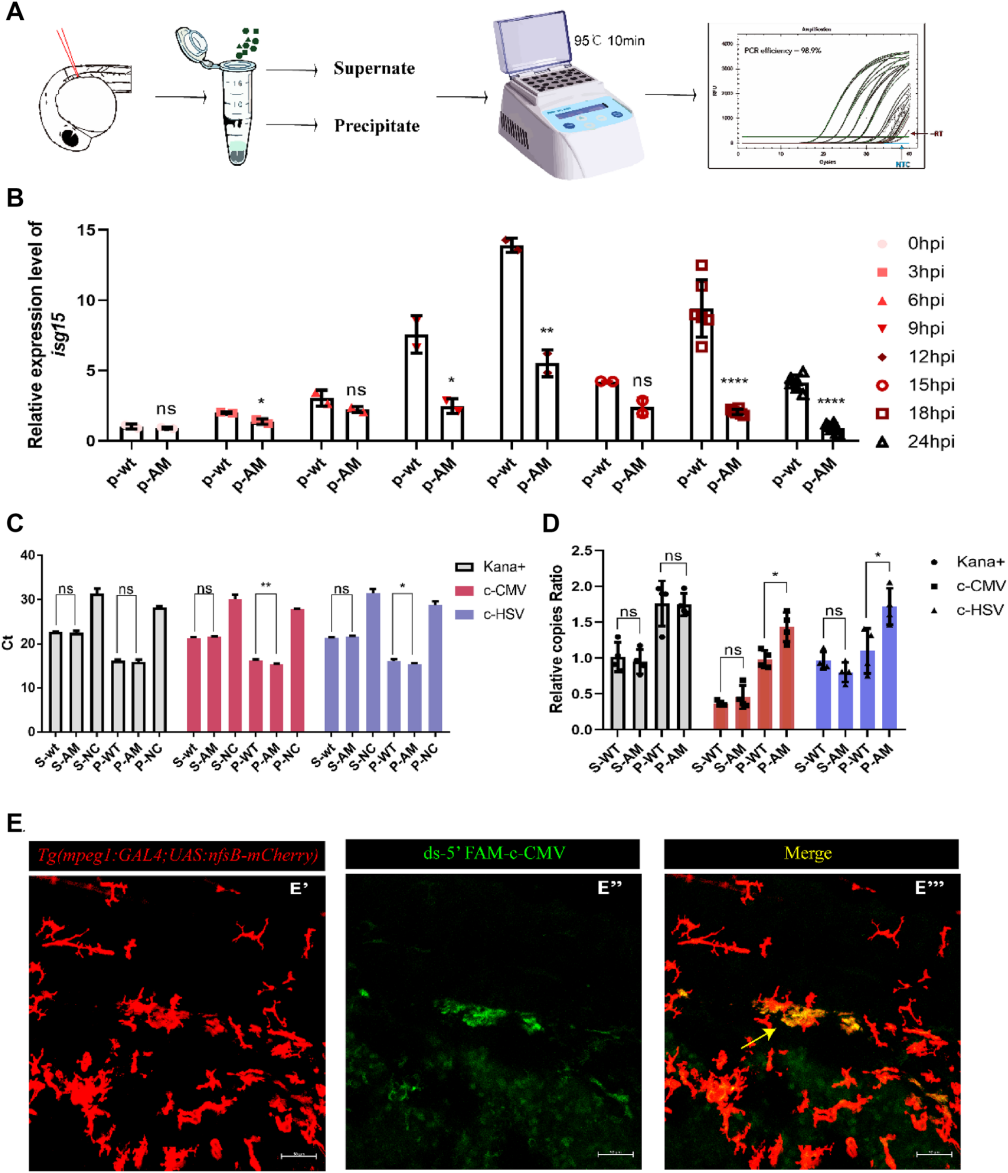
Macrophages recognize the core sequence to allow swallowing and digestion of invading plasmids. **A** Microinjection of plasmids into the central artery of zebrafish embryos. **B** qRT-PCR results showing reduced expression of *isg15* 12 h-post-injection when the core sequences in pEGFP-N1 are mutated. **C**, **D** Q-PCR results indicating that pEGFP-N1 was digested more rapidly than all mutant forms of pEGFP-N1. **E** Confocal scanning image showing macrophages swallowing free DNA fragments in zebrafish

The possibility arises that invasion of a multicellular organism by foreign plasmid DNA may stimulate a similar immune response. The zebrafish immune system is similar to that of mammals and in vitro fertilization of zebrafish is easily manipulated [9]. Following injection of pEGFP-N1 into the central artery of zebrafish embryos at 36 hpf and inspection over 12–24 h-post-injection (hpi), it was observed that macrophages can swallow free plasmids in the circulatory system (Fig. 4E). Levels of macrophage *isg15* expression showed a trend of first increasing and then decreasing (Fig. 4B). However, mutation of the pEGFP-N1 sequence caused a significant reduction in the macrophage response (Additional file 1: Fig. S5A–H). Embryos were collected after 12 hpi, cut into pieces with scissors to release macrophages in the circulatory system, centrifuged and DNA extracted from the precipitate. Q-PCR was used to quantify the pEGFP-N1 plasmid in the precipitated DNA fraction (Fig. 4A). No difference was detected in the kana site, which encodes kanamycin as a control. However, for the group injected with the mutated plasmids, the Ct value of c-CMV and c-HSV was significantly reduced. These results demonstrate that the mutated plasmid has a long half-life in the cell whereas the plasmid containing the core sequence will be degraded quickly (Fig. 4C, D).

## The plasmid containing the core sequence causes the defense response, leading to rapid degradation in *Escherichia coli*

**Fig. 5:**
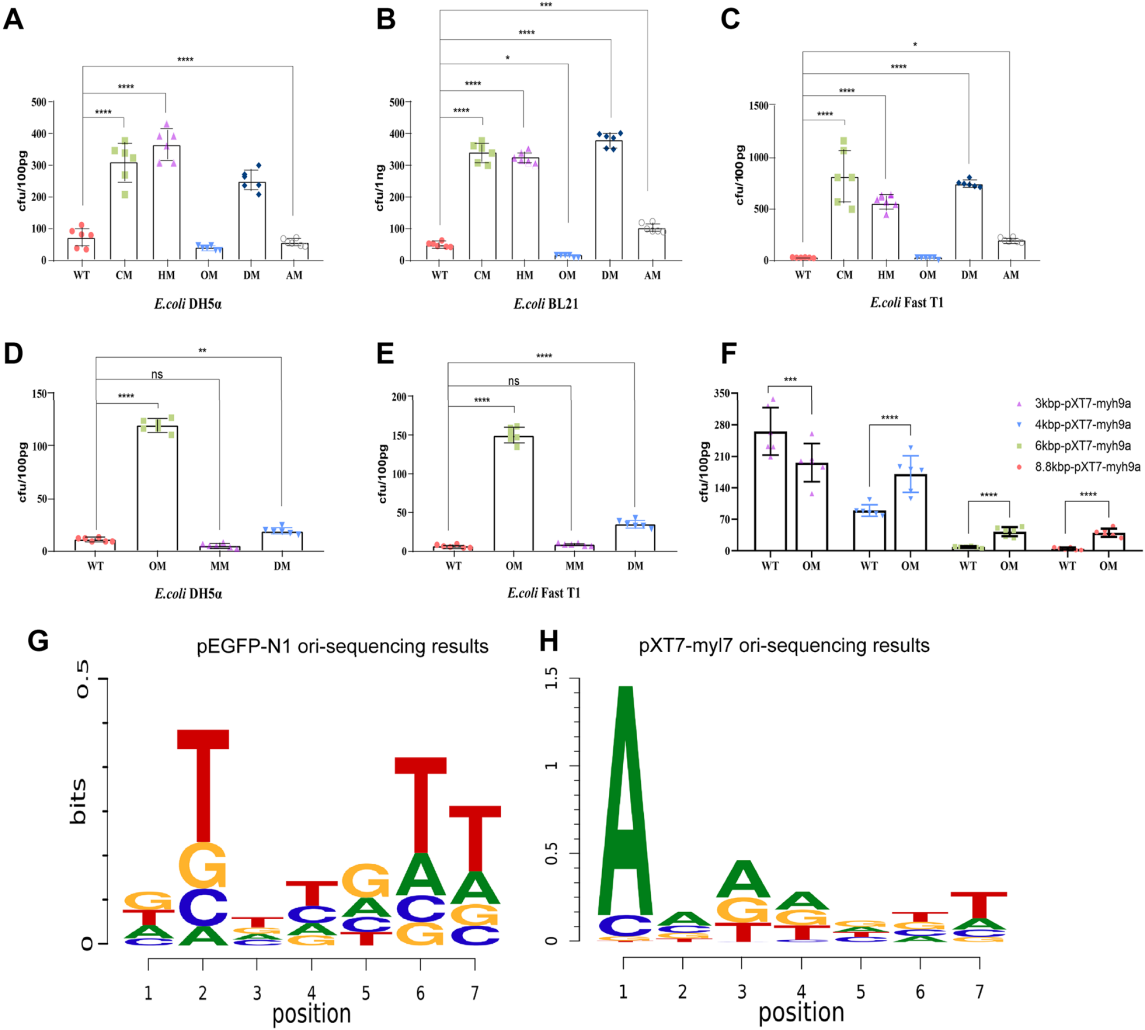
Core sequences protect prokaryotic cells from plasmid infection and mutation of the core sequences increases plasmid transformation efficiency. Transformation efficiencies of different mutant forms of pEGFP-N1 in *E. coli* DH5α (**A**), *E. coli* BL21 (**B**), and *E. coli* FastT1 (**C**). Transformation efficiencies of different mutant forms of pXT7-myh9a in *E. coli* DH5α (**D**) and *E. coli* FastT1 (**E**). **F** Transformation efficiencies of different sized forms of pXT7-myh9a with mutant core sequence in ori. When the core sequence in ori is mutated, the number of clones is reduced for the small size pXT7-myh9a but increased for the large plasmid. Sanger sequencing results showing that the ori sequence of pEGFP-N1 favors survival compared with the 5ʹ-GTTTGTT-3ʹ sequence (**G**), but the ori sequence of pXT7 my17 confers no survival advantage (**H**)

Mutant plasmids were constructed with a single core sequence (CM, HM, OM), a double mutant plasmid with simultaneous mutations of c-CMV and c-HSV and a plasmid with all three sites mutated (AM) (Additional file 1: Fig. S5A–C)5. In three different strains of competent *E. coli* cells, DH5α, FastT1 and BL21, the transformation efficiency of plasmids CM, HM and DM were significantly higher compared with the unmutated plasmids (Fig. 5A–C). The transformation efficiency of the OM plasmid was not significantly different from the unmutated plasmid (Additional file 1: Fig. S5K). These results indicate the presence of a host defense effect that depends on pEGFP-N1 containing the core sequence in *E. coli* cells.

Considering the *E. coli* DH5α strain as an example, 100 pg, 10 pg and 1 pg of plasmid pEGFP-N1 were transformed into the same volume of competent cells. For100 pg plasmid there was a marked effect but for 10 pg or 1 pg plasmid, too few transformed clones were produced, resulting in a large variation in transformation efficiency (Additional file 1: Fig. S5I, J).

Another plasmid, pXT7-myh9a, with a lower transfection efficiency was selected. The plasmid contained two core sequences, one in ori and the other in the *myh9a* coding sequence. The core sequences at these two sites were mutated according to the previous strategy and plasmids with an ori mutation, *myh9a* mutation or with both sites mutated were constructed (Additional file 1: Fig. S5L). Transformation efficiency was increased by 1119% ((119.5–10.67)/10.67 = 11.19) when the core sequence in ori was mutated in the OM plasmid (Fig. 5D, E). By contrast, mutating the core sequence in *myh9a* derived from the zebrafish genome had no effect on the transformation efficiency of the plasmid. We conclude that the core sequence within ori is essential for resisting plasmid invasion but core sequences within intrinsic coding regions have no impact on the defense response in cells.

The impact of plasmid size on the differences in transformation efficiency of mutations in ori were investigated. Plasmids containing the same mutations as DM and MM were constructed and the length of *myh9a* truncated to produce plasmids of different sizes: 3 kbp (3027 bp), 4 kbp (4103 bp), 6 kbp (6294 bp) and 8.8 kbp (8913 bp). We found that the number of transformed clones of the 3 kbp unmutated plasmid (224.5 ± 52.6) was not significantly higher than that of the mutant plasmid (193 ± 42.6). However, the number of transformed clones of the unmutated 4 kbp plasmid was 89.5 ± 11.5and increased significantly to 170.8 ± 37.1, with a growth rate of 90.9%, when ori was mutated. Moreover, the growth rate of the 6 kbp plasmid increased by 384% ((42.8–8.8)/8.8) after mutation and that of the 8.8 kbp plasmid increased by 738% ((40.5–4.8)/4.8) (Fig. 5F). These results indicate that the core sequence improves transformation efficiency for large plasmids and also indicates the role that the core sequence plays in protecting cells from foreign plasmid invasion.

The sequence of 7 bases in the core sequence position of ori in different sized plasmids was investigated by randomly annealing bases at seven core positions, putting the sequences into the pEGFP-N1 and pXT7-myl7 plasmids before adding ampicillin for resistance screening. After 4 h, the plasmids were extracted for deep sequencing of ori. The results show that the core sequence, 5ʹ-GTTTGTT-3ʹ, accounted for 19.06% of the total of 371 sequences in pEGFP-N1 (Fig. 5G). However, for the pXT7-myl7 plasmid, 5ʹ-GTTTGTT-3ʹ only accounted for 2.24% of a total of 501 sequences and no influence was shown of the position of the core sequence (Fig. 5H). The above data shows that for larger plasmids, the core sequence 5ʹ-GTTTGTT-3ʹ is not conducive to the survival of the plasmid but may become the target of attack. Mechanisms involved in the host defense against the core sequence merit further study.

Moreover, we have shown that the ds-CMV fragment with the core sequence was cleaved when delivered to the zebrafish zygote, indicating that the core sequence is recognized and attacked by the defense system of eukaryotic cells. In addition, transcriptome sequencing analysis showed that the transcription level of *E.coli* DH5α transformed by c-CMV or by the c-CMV mutant, pEGFP-N1, was altered. Thus, we suspect that the core sequence may stimulate a new unknown defense response (data not shown). Besides, we also found that the expression of GFP on the plasmid was affected when the core sequence was mutated. In HEK293T cells, compared with cells transfected with pEGFP-N1 (Additional file 1: Fig. S7A–A’’’), photographed using the same parameters, it was found that HM (Additional file 1: Fig. S7C–C’’’) and OM (Additional file 1: Fig. S7D–D’’’) transfected cells had no GFP fluorescence, while DM (Additional file 1: Fig. S7E–E’’’) and AM (Additional file 1: Fig. S7F–F’’’) transfected cells showed attenuation of the fluorescence intensity. But in *E. coli*, the mutation of the core sequence does not affect the yield of the plasmid (Additional file 1: Fig. S6K). This result indicates that the mutation of core sequence will induce different defense mechanisms and affect the activity of different elements in the plasmid in eukaryotic cells. Our findings merit further study to illuminate the mechanism responsible.

To summarize, core sequences present in the CMV promoter, HSV poly (A) signal and ori are central to the resistance of bacteria to infection by foreign plasmids or DNA fragments. Moreover, such mechanisms may be exploited to improve techniques of transformation efficiency for large plasmids.

## Supplementary Information

**Additional file 1**.

## Data Availability

All data generated or analyzed during this study are included in this published article [and its Additional files]. Series record GSE165422 of transcriptome sequencing results is an open-source collaborative initiative available in the GEO repository (https://www.ncbi.nlm.nih.gov/geo/info/linking.html).

## Abbreviations

RM: Restricted modification
pAgo: The prokaryotic Argonaute system
H3K4me3: Tri-methylated lysine of histone H3
hpf: Hour-post-fertilization
PTC: Premature stop codons
