# Supplementary material for: Both prokaryotes and eukaryotes produce an immune response against plasmids with 5ʹ-GTTTGTT-3ʹ
Source: Cell Biosci. 2022 Jun 11;12:87. doi: 10.1186/s13578-022-00825-3 (PMC9188160; doi:10.1186/s13578-022-00825-3)
Supplement: Supplementary file 1 — Additional file 1. [file 13578_2022_825_MOESM1_ESM.docx]

**Supplementary data**

**Methods**

**Zebrafish husbandry**

Zebrafish are raised in a circulating water system (China, ESEN) at a room temperature of 28.5°C, with fixed light for 14 hours a day and 10 hours in the dark. Zebrafish are fed shrimp (Salt Creek, USA) every morning and afternoon. Wild-type Tubingen strain (TU), *Tg (mpeg1: Gal4/UAS: nfsB-mCherry)* strain was used in this study.

**Cell culture and Transfection**

HCT116 and HEK293T cell lines (ATCC, USA) were grown in high-glucose Dulbecco's modified Eagle's medium (DMEM) (Gibco, USA, 12100046) supplemented with 10% fetal bovine serum (PAN, German) and 1% penicillin/streptomycin (Gibco, USA, 10378016) under 5% carbon dioxide (CO2) at 37°C. Cells were plated on 60-mm dishes at a density of 1 × 10^6^ cells per dish to reach about 70% confluency. And cells were transfected with plasmids or DNA fragments using Lipofectamine 3000 (Invitrogen, USA, L3000015) according to the manufacturer’s protocol.

**Transcriptome sequencing and data analysis**

In the 1-cell stage of zebrafish embryos, 8ng/μL pEGFP-N1 was injected, and the embryos of the injected group and the non-injected group were collected when the embryos developed to 1hpf, 6hpf and 12hpf. Add 1mL TRIzol (Invitrogen, USA, 15596018), store at -80℃, and transport to Shanghai Liebing Biomedical Technology Co., Ltd. for transcriptome sequencing analysis. The sequencing data named GSE165422 has been uploaded to NCBI. According to the results of transcriptome sequencing, the differential genes related to self-defense such as immune response were screened, and a heat map was made using GraphPad Prism 8 (GraphPad Software, La Jolla, CA).

Related GO analysis produced GO chord diagrams and motif logo were plotted by http://www.bioinformatics.com.cn, an online platform for data analysis and visualization.

**Plasmid construction**

The core sequence in the CMV promoter region, HSV poly(A) signal and ori region in the pEGFP-N1 plasmid was mutated, and 5’-GTTTGTT-3’ was mutated to 5’-CAAACAA-3’. Design primers and use overlapping PCR and ABclonal MultiF Seamless Assembly Mix (RK2102, ABclonal, China) to construct c-CMV mut plasmids with mutations in the core sequence of the CMV promoter region, c-HSV mut plasmids with mutations in the core sequence of the HSV poly(A) signal region, and c-ori mut plasmid with mutations in the core sequence of ori. Following the same method, the envelope protein expression vectors pCMV-VSV-G and pLVX-IRES-Zsgreen1 were successfully constructed for the production of lentivirus and MuLV retroviral particles. The plasmids with all the core sequences in the backbone of pLVX-IRES-Zsgreen1 were successfully constructed. Similarly, the core sequence of ori and myh9a coding sequence in plasmid pXT7-myh9a was mutated. And constructed plasmids of different sizes, including 3kbp, 4kbp, 6kbp and 8.8kbp, of which the plasmid only has a core sequence on ori, except for myh9a. See Table S1 for specific primer sequences.

In order to understand the sequence of the ori core site on pEGFP-N1 and pXT7-myl7, the core sequence was synthesized randomly by Genscript (China). Then the sequence was recombined with vectors by using ABclonal MultiF Seamless Assembly Mix (RK2102, ABclonal, China). The plasmids were extracted and delivered to Sequen bio-info Studio for Next-generation sequencing (NGS), and the sequencing results was analyzed by dada2.

**RNA analysis and qRT–PCR**

For qRT–PCR, Total RNAs were extracted from at least 30 embryos for each one group, by using Direct-zol RNA MiniPrep Kit (ZYMO Research, USA, R2050) following the manufacturer protocol. qRT‐PCR were performed by using HiScript 1st stand cDNA synthesis kit (Vazyme, NanJing) kit and SYBR Green (A25742, Roche, Switzerland) method following their manufacturer protocol. The relative expression levels of test genes were normalized by the expression of *gapdh* in zebrafish and *GAPDH* in mammalian cells. See Table S2 for specific primer sequences.

**Chromatin immunoprecipitation-PCR**

Exogenous DNA fragments ds-CMV-WT, ds- CMV-Mut, and plasmid pEGFP-N1, c-CMV mutated pEGFP-N1 injection concentration is 100nM, each group is injected with 1000 embryos, and then all these embryos are collected at 3hpf for ChIP assay. The ChIP-PCR assay was performed using the EZ-ChIP Chromatin Immunoprecipitation Kit (Millipore, USA, 17-371) following the manufacturer’s instructions. 1 μg of Anti-H3K4me3 polyclonal antibody (Abcam, USA, ab8580) was used to immune-precipitate all DNA, and 1 μg of mouse IgG was used as negative control. The semiquantitative PCR was performed under the condition of 95 oC, 5 min; 25X (95 oC 30 s; 52 oC, 30 s; 72 oC, 15 s); 72 oC, 7 min. The PCR products were then subjected to separation by 2% agarose electrophoresis. The primers used for ChIP-PCR and qRT-PCR are listed in Table S2.

**Plasmid transformation efficiency test**

100 pg of pEGFP-N1, pEGFP-N1 (CM, HM, OM) mutated at three sites respectively, pEGFP-N1 (DM) mutated at the same time as c-CMV and c-HSV, and the pEGFP-N1 (AM) with simultaneous mutation at the site was transformed into 50μL *E.coli* DH5α competent cells (Vazyme, China, C502-02). The same operation was performed on competent strains *E.coli* FastT1 (Vazyme, China, C505-02)and *E.coli* BL21 (Vazyme, China, C504-02). Place the LB plate upside down in a 37°C bacterial incubator for 12-16 hours, and then count the bacterial clones on the plate. Each sample was transformed six times independently.

**Q-PCR detection of the relative content of plasmids in *E. coli* strains**

10ng pEGFP-N1 and pEGFP-N1 DM plasmids were transformed into 100μL *E.coli* DH5α competent cells, centrifuged at 8,000g for 1min before activation, and the supernatant was removed. Add 10 mL of LB medium to the cell pellet, mix by pipetting carefully, and place it in a shaker at 37°C for culture. Take 30 minutes as the starting point for sampling, measure the value of bacterial OD600 before sampling and adjust to the same. Take the same volume of bacterial solution, centrifuge at 12,000g for 1min, remove the supernatant, add 50μL of 1xTE buffer and vortex to completely resuspend the bacterial pellet, then add 50μL of phenol chloroform, vortex for 15s, centrifuge at 12,000g for 5min, take out 40μL of supernatant Put it in the eight tubes for use. Next, samples were taken every 30 minutes, and the crude bacterial genome was prepared according to the same operation. The PCR reaction system is prepared on ice. Each reaction well has a volume of 10μL, containing 5μL 2x PowerUp SYBR Green Master Mix (A25742, ABI, USA), 0.3μL 10μM forward primer, 0.3μL 10μM reverse primer, 3.4μL Nuclease- free Water, and finally add 1μL of genome template. The reaction program is 50°C 2 minutes, 95°C 2 minutes, 40 cycles (95°C 3 seconds, 60°C 1 minute). Use kana-F1/R1 as internal reference primer. Primers are listed in Table S1.

**Microinjection of plasmids and DNA fragments into the central artery of zebrafish larvae and embryos**

In order to detect the response to foreign plasmids or nucleic acid fragments in the early stage of embryos, both plasmids and nucleic acid fragments were diluted to a concentration of 2.6nM, and then injected into the extreme junction of animals and plants during the 1-cell stage of zebrafish. The injection volume of each embryo was 2nL. In order to detect whether macrophages would swallow foreign nucleic acid plasmids or fragments, 500ng/μL pEGFP-N1 and pEGFP-N1 AM, 20μM ds 5'-FAM-CMV and ds 5'-FAM-CMV-mut were injected into the growth respectively. The central artery of zebrafish juveniles up to 96hpf, each juvenile was injected with a volume of 2nL. In the above injection experiment, Hank' solution was injected as a blank solvent control group, and uninjected embryos were used as a negative control group. 24 hours after injection, 100 embryos were collected from each group, washed with 1xPBS three times, and drained of liquid. Then add 200μL of 1xPBS to each group, put the juveniles on ice after anesthesia for 10 minutes, when the juveniles die, immediately cut the tissue with ophthalmic scissors to expose the cells in the circulatory system. The suspension was filtered with a 100 μm cell strainer, and the filtrate was centrifuged at 1,000 rpm for 5 min. Put the supernatant under a microscope to observe whether there are cells, then separate the filtrate and the precipitate into a 1.5 mL centrifuge tube, and add 1 mL TRIzol for RNA extraction. On the other hand, the same number of juvenile fish were subjected to the same operation as above, and the filtrate was centrifuged at 12,000 g for 2 minutes. Put the supernatant under a microscope to observe whether there are cells. If there are, centrifuge at an increased speed. Then remove the supernatant, add 30μL of 1xPBS to the pellet, mix by pipetting, and then put it in a 95℃ water bath for 10 minutes, lysing the cells to expose the genome, centrifuge at 12,000g for 5min, collect the supernatant as a QPCR template and store it at -20℃.

**Western Blot**

Cultured HEK 293T cells were lysed in RIPA buffer (Thermo Scientific, Waltham, MA, USA). Protein was quantified with Bradford Dye (Bio-Rad 500-0006), and 20 μg was loaded onto an SDS-polyacrylamide gel (SDS-PAGE) with SDS/Tris/Glycine running buffer. The antibodies used for blotting were: 1:5000 H3K4me3 (Abcam ab8580), 1:2000 GAPDH (Abcam ab9485). Membranes were incubated in primary antibody in 0.1% TBST with 5% milk at 4 °C overnight. Membranes were incubated in 1:2000 secondary antibody in 0.1% TBST for 1 h and then imaged on Tanon-5200 Chemiluminescent Imaging System (Tanon Science & Technology) with 3–5 blots assessed for each experiment.

**Imaging**

All confocal images were acquired using a Zeiss LSM880 confocal microscope.

**Statistics**

Experiments were performed two or three times independently. Data are shown as mean ± S.D. Statistical analysis was carried out with GraphPad Prism 7 (GraphPad Software, La Jolla, CA). Data were firstly tested for normality using the Kolmogorov–Smirnov’s test. If data sets exhibit normal distribution, we employed student’s T-test for equal variances or Welch’s T-test for unequal variances. If data sets are found not to exhibit normal distribution, Mann-Whitney test was applied. A value of p < 0.05 (*) was considered statistically significant, and p < 0.01 (**), p < 0.001 (***) were considered statistically very significant.

Supplementary Materials

Figures. S1 to S7

Tables S1 to S2


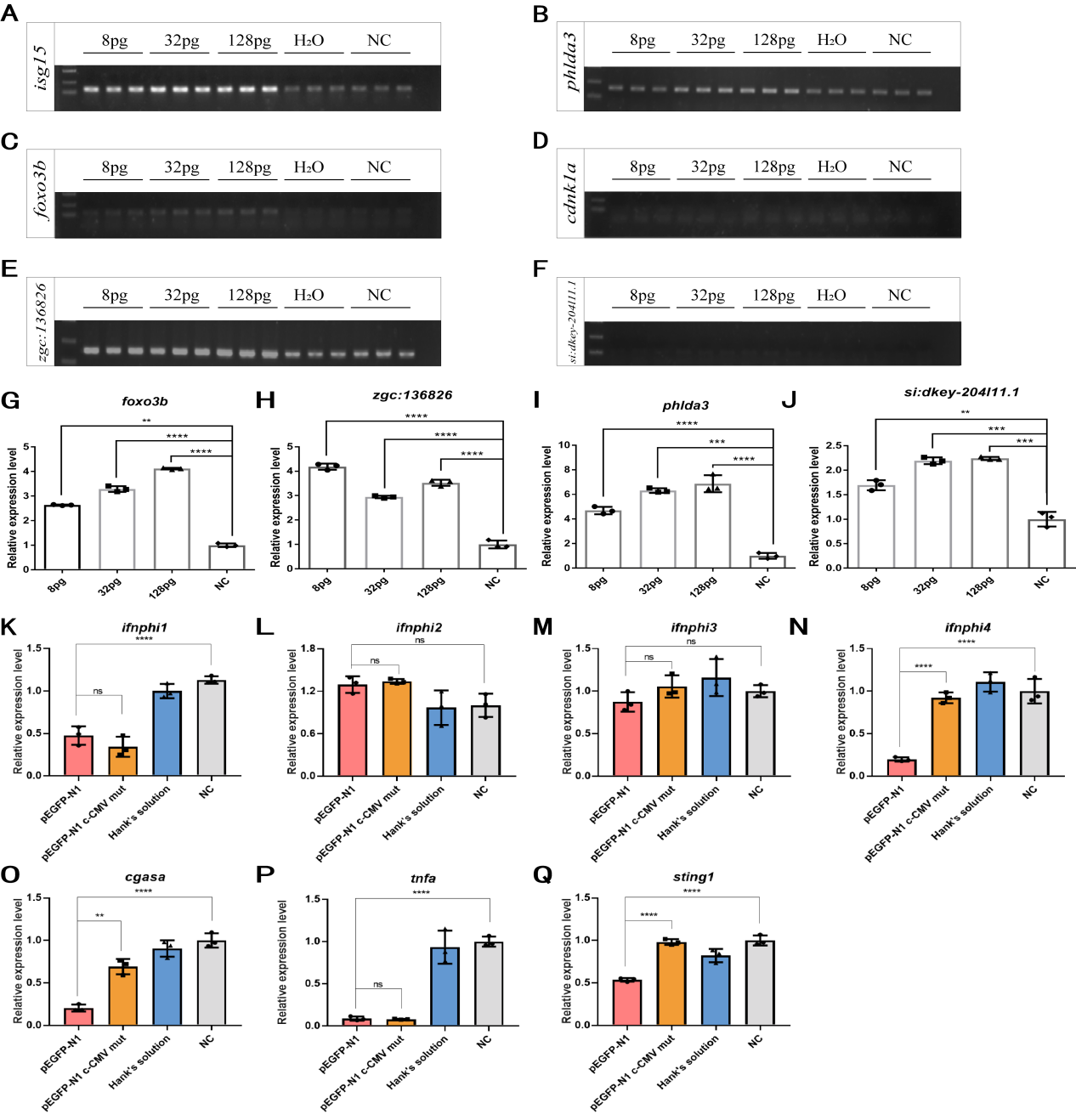


Figure. S1. Up-regulation of genes related to the endogenous immune response and apoptosis. Semi-quantitative PCR verified that when different concentration gradients were injected into 1-cell stage zebrafish embryos, the expression levels of *isg15*, *foxo3b*, *zgc:136826*, *phlda3* and *si:dkey-204l11.1* increased significantly. (G-J) The results of qRT-PCR further support this conclusion. The results of qRT-PCR showed that the expression level of IFN-related genes in plasmid-injected embryos, including *ifnphi1* (K), *ifnphi2* (L), *ifnphi3* (M), *ifnphi4* (N), *cgasa* (O), *tnfa* (P) and *sting1*(Q). **, *p* < 0.01; ***, *p* < 0.001; ****, *p* < 0.0001. ns, no significance (*p* > 0.05). Results are shown + S.D. MW: DL5000 DNA Molecular Weight Marker.


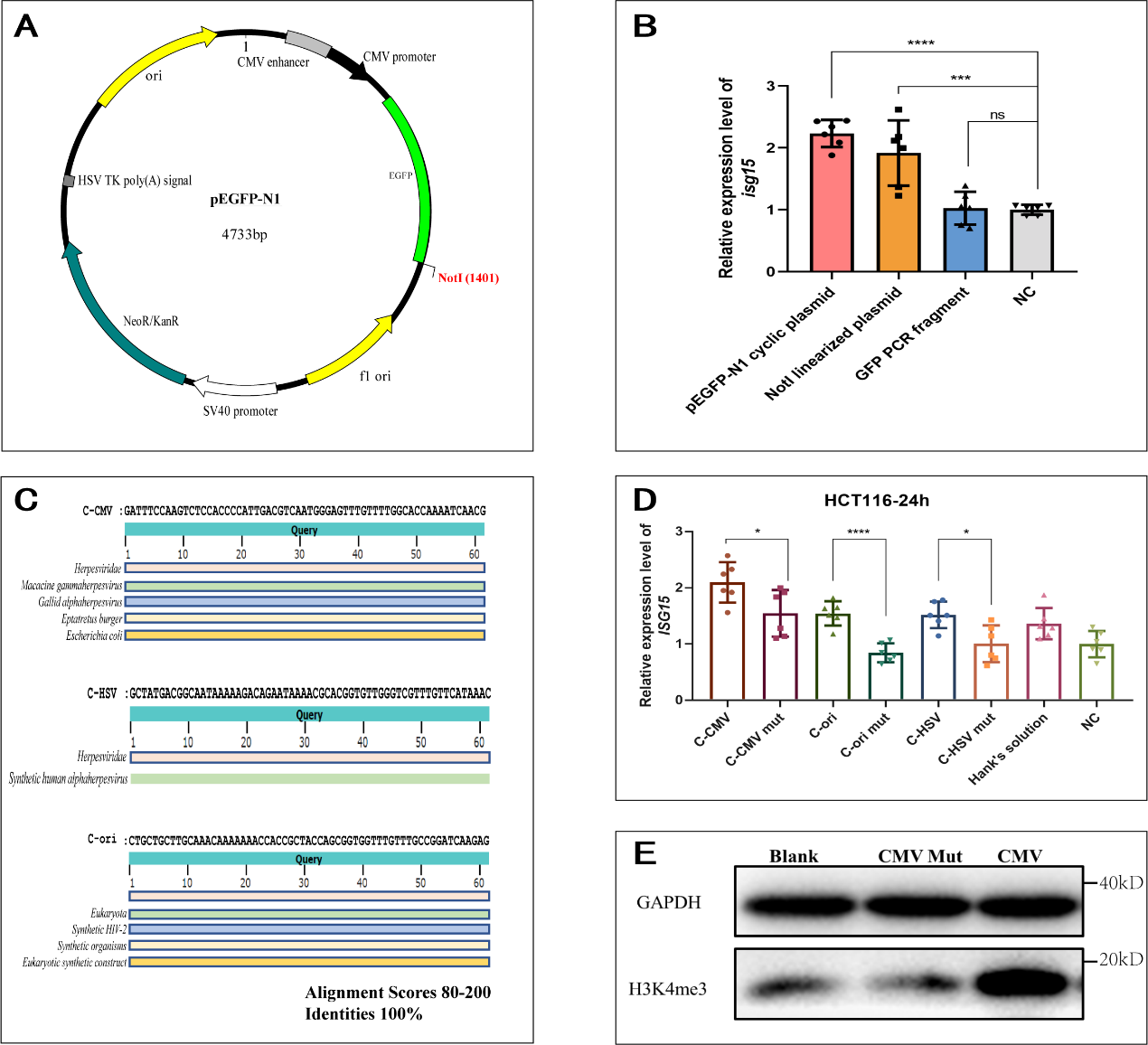


Figure. S2. Three fragments on the plasmid backbone originate from the genome of viruses and bacteria and act as epigenetic regulators binding to the promoter of*isg15* after entering eukaryotic cells. (A) The detailed map of pEGFP-N1. (B) the NotI digested liner pEGFP-N1, EGFP fragment and circular form were injected into zebrafish zygotes. Embryos were collected at 3 hpf for qRT-PCR. (C) Alignment of the c-CMV, c-HSV and c-ori fragment sequences in the database showing that the three fragments all originate from bacteria and viruses. (D) The results of qRT-PCR indicate that the three fragments activate *isg15* expression in HEK 293T cells. Activation is weakened when the core sequence is mutated. (E) In the HEK 293T cell line, mutated and unmutated c-CMV pEGFP-N1 were transfected and histone methylation H3K4me3 level in the cells detected by Western blotting. Activation and up-regulation of *isg15* after injection with unmutated CMV is shown. The level of histone methylation H3K4me3 in the promoter region. *, *p* < 0.05; **, *p* < 0.01; ***, *p* < 0.001; ****, *p* < 0.0001. ns, no significance (*p* > 0.05). Results are shown + S.D.


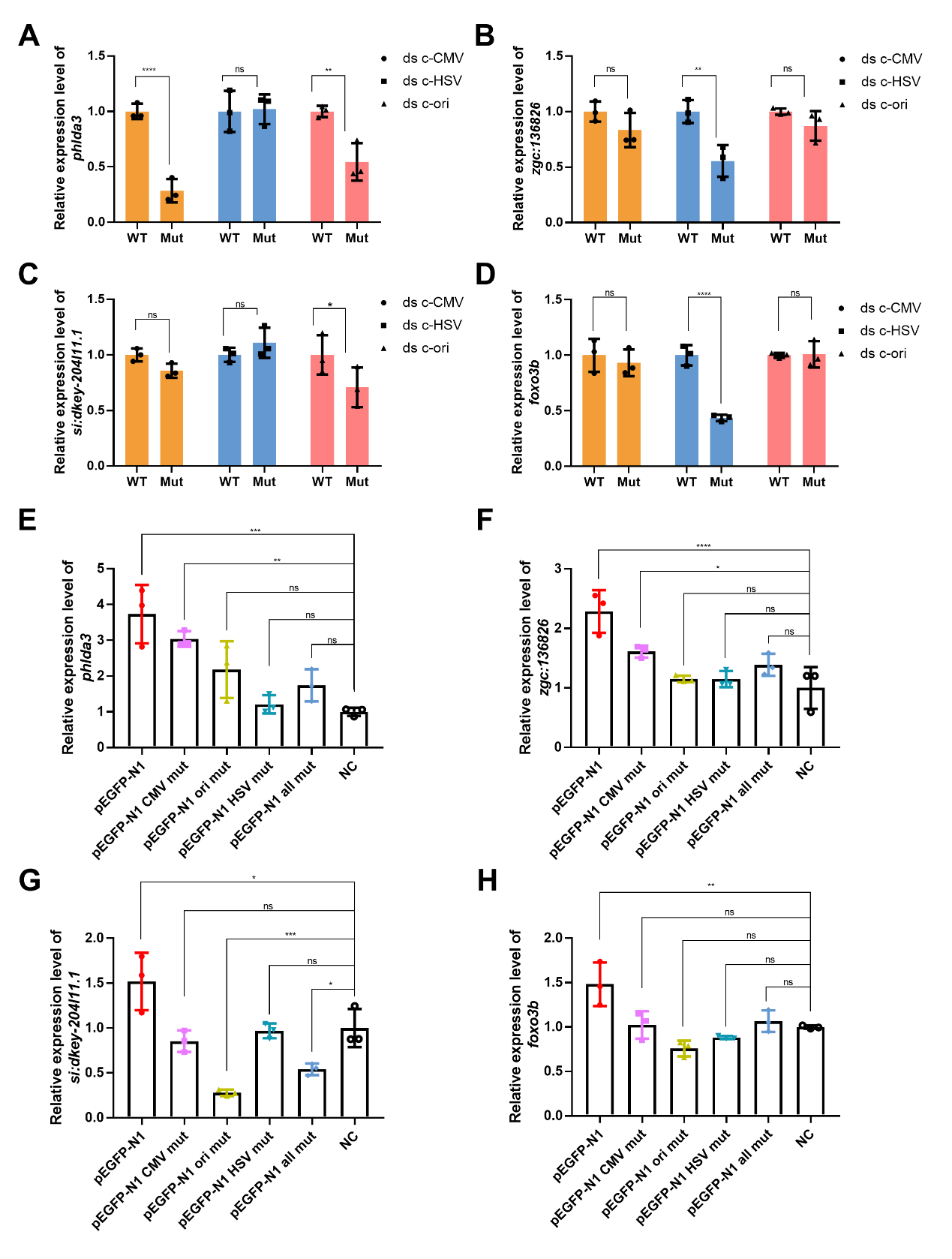


Figure. S3. Other four genes are not fully response to the DNA fragments or plasmids with core sequence. (A) qRT-PCR result shows the expression level of *phlda3* in different DNA fragments groups. (B) qRT-PCR result shows the expression level of *zgc:136826* in different DNA fragments groups. (C) qRT-PCR result shows the expression level of *si:dkey-204l11.1* in different DNA fragments groups. (D) qRT-PCR result shows the expression level of *foxo3b* in different DNA fragments groups. (E) qRT-PCR result shows the expression level of *phlda3* in different plasmids groups. (F) qRT-PCR result shows the expression level of *zgc:136826* in different plasmids groups. (G) qRT-PCR result shows the expression level of *si:dkey-204l11.1* in different plasmids groups. (H) qRT-PCR result shows the expression level of *foxo3b* in different plasmids groups. *, *p* < 0.05; **, *p* < 0.01; ***, *p* < 0.001. ns, no significance (*p* > 0.05). Results are shown + S.D.


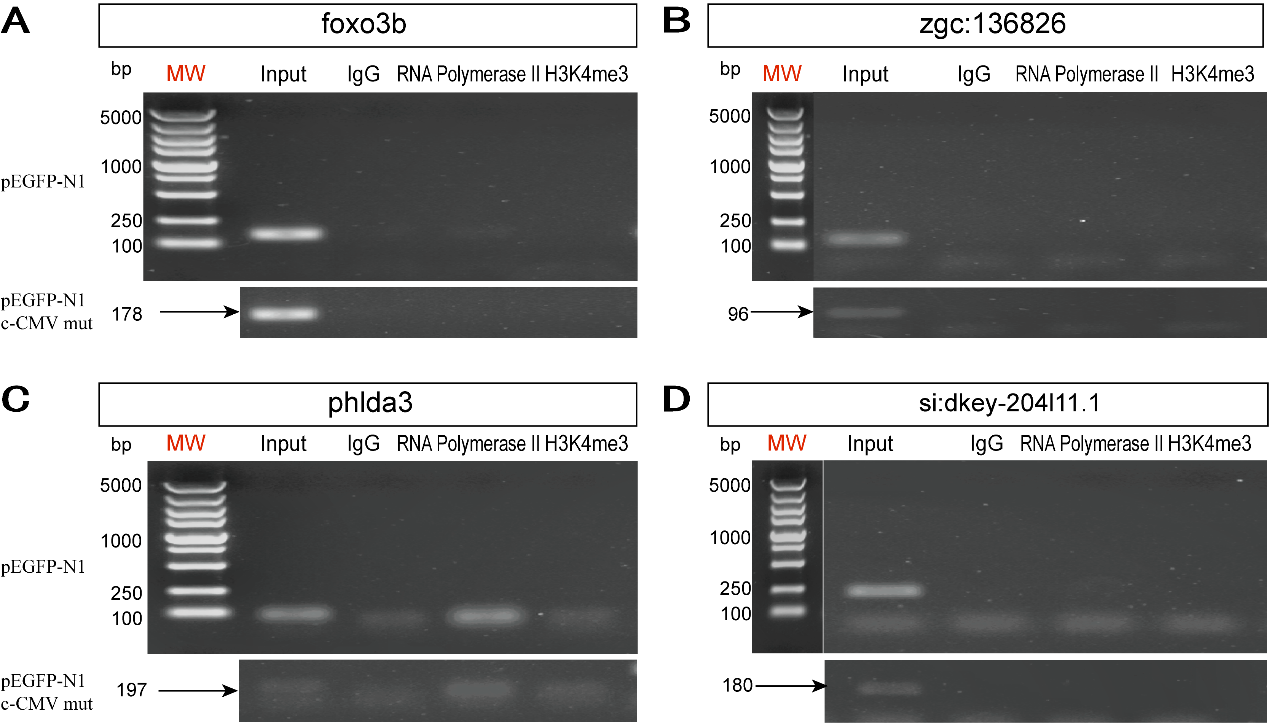


Figure S4. There were no significant changes in the H3K4me3 methylation levels in the promoter regions of the other four genes. (A) The results of semi-quantitative PCR showed that there was no significant change in the methylation level of H3K4me3 in the *foxo3b* promoter region. (B) The results of semi-quantitative PCR showed that there was no significant change in the methylation level of H3K4me3 in the *zgc:136826* promoter region. (C) The results of semi-quantitative PCR showed that there was no significant change in the methylation level of H3K4me3 in the *phlda3* promoter region. (D) The results of semi-quantitative PCR showed that there was no significant change in the methylation level of H3K4me3 in the *si:dkey-204l11.1* promoter region. MW: DL5000 DNA Molecular Weight Marker.


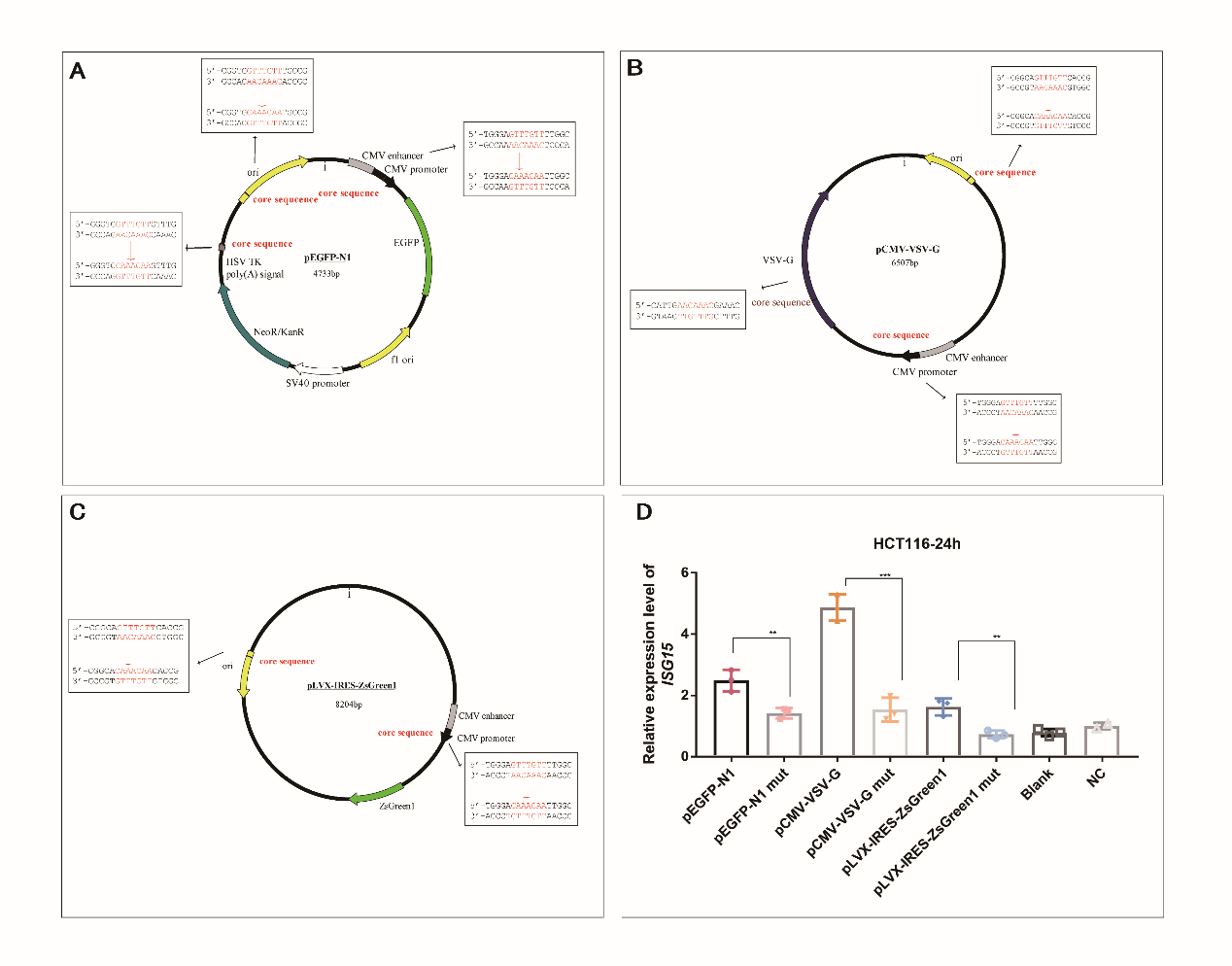


Figure. S5. In eukaryotic cells, plasmids containing core sequences also regulate *isg15* expression epigenetically. (A-C) The detailed mutant sites map of pEGFP-N1, pCMV-VSV-G and pLVX-IRES-ZsGreen1. (D) The pEGFP-N1, pCMV-VSV-G and pLVX-IRES-ZsGreen1 and their mutated forms were transfected into HCT116 cell line. *, *p* < 0.05; **, *p* < 0.01; ***, *p* < 0.001; ****, *p* < 0.0001. ns, no significance (*p* > 0.05). Results are shown + S.D.


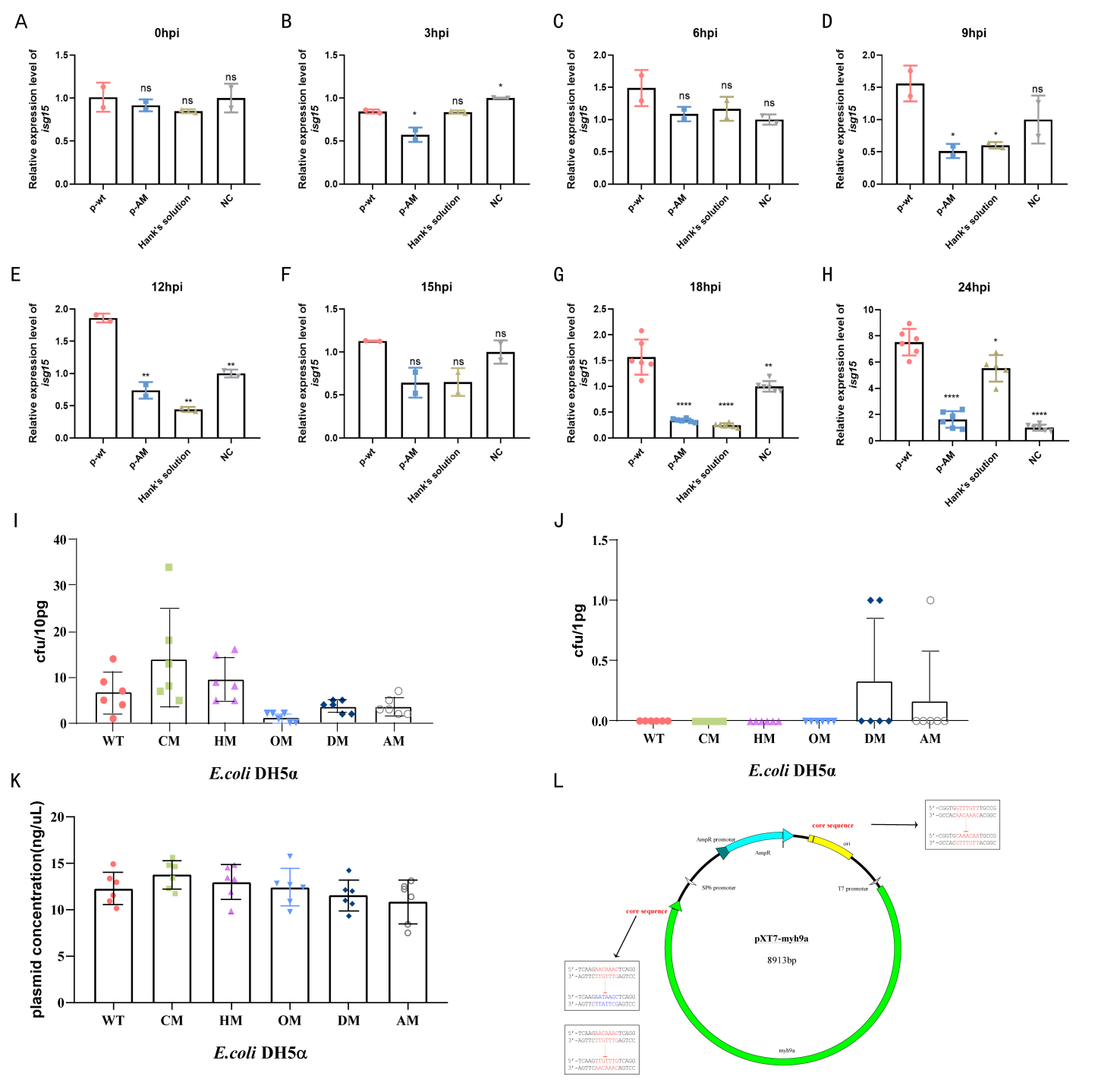


Fig. S6. The presence of the core sequence helps prokaryotic cells resist plasmid invasion but does not affect plasmid replication. The results of qRT-PCR show activation of *isg15* expression after injection with all mutant core sequences in pEGFP-N1. (I) The transformation efficiency of 10pg pEGFP-N1 in *E.coli* DH5α. (J) The transformation efficiency of 10pg pEGFP-N1 in E.coli DH5α. (K) The concentration of different mutant pEGFP-N1. (L) The detailed mutant sites map of pXT7-myh9a. *, *p* < 0.05; **, *p* < 0.01; ***, *p* < 0.001; ****, *p* < 0.0001. ns, no significance (*p* > 0.05). Results are shown + S.D.


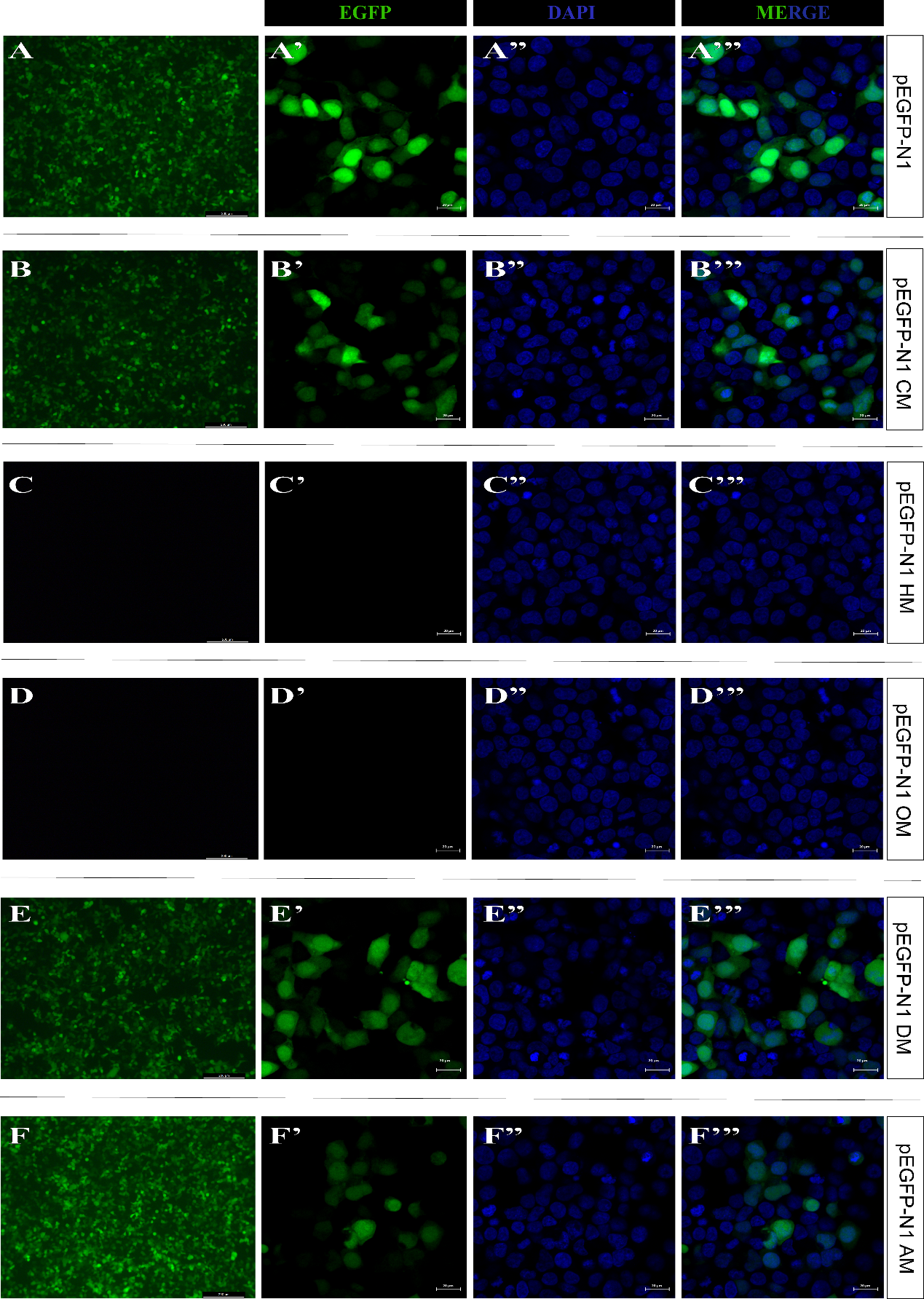


Figure S7. When the core sequence on CMV, HSV and ori is mutated, it will affect the expression of GFP. Compared with pEGFP-N1 (A-A’’’), GFP expression was impaired after transfection of HM (C-C’’’) or OM (D-D’’’) plasmid, and the intensity of GFP expression was reduced on DM (E-E’’’) and AM (F-F’’’) plasmids, but not on CM (B-B’’’) plasmid.

*Supplementary Table 1. Primers used in plasmids construction in the study.*

| **Name** | **GenBank Accession numbers** | **Sequence** | **Amplicon Size (bp)** | **Tm (℃)** | **Notes** |
| --- | --- | --- | --- | --- | --- |
| CMV-WT-F1 | U55762 | GATTTCCAAGTCTCCACCCCATTGACGTCAATGGGAGTTTGTTTTGGCACCAAAATCAACG | 61 | 93.4 | For synthesis double-strand DNA fragment |
| CMV-WT-R1 |  | CGTTGATTTTGGTGCCAAAACAAACTCCCATTGACGTCAATGGGGTGGAGACTTGGAAATC |  | 93.4 |  |
| ori-wt-F1 | U55762 | CTGCTGCTTGCAAACAAAAAAACCACCGCTACCAGCGGTGGTTTGTTTGCCGGATCAAGAG | 61 | 95.5 |  |
| ori-wt-R1 |  | CTCTTGATCCGGCAAACAAACCACCGCTGGTAGCGGTGGTTTTTTTGTTTGCAAGCAGCAG |  | 95.5 |  |
| HSV-wt-F1 | U55762 | GCTATGACGGCAATAAAAAGACAGAATAAAACGCACGGTGTTGGGTCGTTTGTTCATAAAC | 61 | 88.9 |  |
| HSV-wt-R1 |  | GTTTATGAACAAACGACCCAACACCGTGCGTTTTATTCTGTCTTTTTATTGCCGTCATAGC |  | 88.9 |  |
| CMV-mut-F1 | U55762 | GATTTCCAAGTCTCCACCCCATTGACGTCAATGGGACAAACAATTGGCACCAAAATCAACG | 61 | 93.9 |  |
| CMV-mut-R1 |  | CGTTGATTTTGGTGCCAATTGTTTGTCCCATTGACGTCAATGGGGTGGAGACTTGGAAATC |  | 93.9 |  |
| ori-mut-F1 | U55762 | CTGCTGCTTGCAAACAAAAAAACCACCGCTACCAGCGGTGCAAACAATGCCGGATCAAGAG | 61 | 95.9 |  |
| ori-mut-R1 |  | CTCTTGATCCGGCATTGTTTGCACCGCTGGTAGCGGTGGTTTTTTTGTTTGCAAGCAGCAG |  | 95.9 |  |
| HSV-mut-F1 | U55762 | GCTATGACGGCAATAAAAAGACAGAATAAAACGCACGGTGTTGGGTCCAAACAACATAAAC | 61 | 88.9 |  |
| HSV-mut-R1 |  | GTTTATGTTGTTTGGACCCAACACCGTGCGTTTTATTCTGTCTTTTTATTGCCGTCATAGC |  | 88.9 |  |
| Kana-F1 | U55762 | CGATCAGGATGATCTGGACGAAG | 608 | 63.4 | For mutating the core sequence in pEGFP-N1 |
| HSV-mut-R1 |  | CGTTTATGTTGTTTGGACCCAACACCGTGC |  | 76.0 |  |
| HSV-ori-mut-F1 | U55762 | CAAACAACATAAACGCGGGGTTCGGTCCC | 411 | 78.1 |  |
| ori-HSV-mut-R1 |  | CAAACAATGCCGGATCAAGAGCTACCAACTC |  | 74.3 |  |
| ori-mut-F1 | U55762 | CAAACAATGCCGGATCAAGAGCTACCAACTC | 1109 | 74.3 |  |
| CMV-ori-mut-R1 |  | GGTGCCAATTGTTTGTCCCATTGACGTCAATGGGGTGGAGAC |  | 87.8 |  |
| CMV-mut-F1 | U55762 | CAAACAATTGGCACCAAAATCAACGGGACT | 546 | 74.9 |  |
| EGFP-R1 |  | GCCGTCGTCCTTGAAGAAGATG |  | 63.6 |  |
| EGFP-F1 | U55762 | AGCAGCACGACTTCTTCAAG | 467 | 55.5 |  |
| Kana-R1 |  | GGCCACAGTCGATGAATCCAG |  | 62.5 |  |
| bata-VSV-F1 | OF125271 | GGTTGTTGTGCTGTCTCATCATTTTG | 423 | 64.9 | For mutating the core sequence in pCMV-VSV-G |
| VSV-mut-R1 |  | TTCCTTGTTTCCAAACAACAATGCTTTCCTTGC |  | 75.8 |  |
| VSV-M13-F1 |  | TTCCTTGTTTCCAAACAACAATGCTTTCCTTGC | 1932 | 75.8 |  |
| M13/pUC Reverse |  | AGCGGATAACAATTTCACACAGG |  | 61.1 |  |
| ori-F1 |  | CCCTCTTCTCTTATGGAGATCCCTC | 984 | 63.2 |  |
| ori-mut-R1 |  | CAGCGGTGCAAACAATGCCGGATCAAGAGCTAC |  | 81.1 |  |
| ori-Amp-mut-F1 |  | TTGTTTGCACCGCTGGTAGCGGTGGTTTTTTTGT | 1387 | 81.0 |  |
| F1ori-F(R) |  | GTGGACTCTTGTTCCAAACTGG |  | 58.7 |  |
| ori-CMV-F1 |  | CAAACAATTGGCACCAAAATCAACGGGACTTTC | 1180 | 70.6 |  |
| CMV-mut-R1 |  | CAATAGGCCGAAATCGGCAAAATCC |  | 76.2 |  |
| CMV-mut-F1 |  | GGTGCCAATTGTTTGTCCCATTGACGTCAATGGGGTG | 923 | 84.9 |  |
| CMV-VSG-R1 |  | GCAATTCACCCCAATGAATAAAAAGGC |  | 69.1 |  |
| ori-F1 | MH325104 | GAACAGGGACTTGAAAGCGAAAG | 1967 | 73.7 | For mutating the core sequence in pLVX-IRES-Zsgreen1 |
| ori-mut-R1 |  | GGTGCCAATTGTTTGTCCCATTGACGTCAATGGGGT |  | 84.9 |  |
| ori-mut-F1 |  | CAAACAATTGGCACCAAAATCAACGGGACTTTC | 1996 | 77.2 |  |
| ori-R1 |  | GAAGGGACGTAGCAGAAGGACG |  | 62.9 |  |
| CMV-F1 |  | CGGGGAAGCTGACGTCCTTTC | 2100 | 65.6 |  |
| CMV-mut-R1 |  | CAGCGGTGCAAACAATGCCGGATCAAGAGCTA |  | 80.8 |  |
| CMV-mut-F1 |  | TTGTTTGCACCGCTGGTAGCGGTGGTTTTTTTGTT | 2386 | 81.4 |  |
| CMV-R1 |  | CTCTCCTTCTAGCCTCCGCTAGTC |  | 62.5 |  |
| PXT7 ori F2 | XM_005165694  X65305 | ACAATGCCGGATCAAGAGCTACCAACTCTTTTTCCGAA | 2377 | 80.8 | To construct different size pXT7-myh9a containing the mutant core sequence |
| PXT7 ori R |  | CTCTTGATCCGGCATTGTTTGCACCGCTGGTAGCGGTGGTTTT |  | 89.2 |  |
| EcoR1-F1 |  | GTACCGAATTCCTCGAGAGATCTGAT | 3611 | 63.6 |  |
| EcoR1-R1 |  | ATCTCTCGAGGAATTCGGTACCGATCTGCCAAA |  | 77.8 |  |
| myh9a 7K-R |  | CACTAGTGATATCAGATCTCTTACTCAGGAGTTGGCTCGGAC |  | 77.7 |  |
| myh9a 1K-F1 |  | ACTTTGGCAGATCGGTACCGATGAGAGGAAACAGCGGGCTCA | 1000 | 87.9 |  |
| myh9a 3K-F2 |  | ACTTTGGCAGATCGGTACCGGAGTCTGAGGCCAAACAAAAGC | 3000 | 85.9 |  |
| PXT7 5.8K F |  | ACTTTGGCAGATCGGTACCGATGTCTGATGCTGAAAAATT | 5800 | 81.1 |  |
| (5'FAM) CMV-WT-F1 | U55762 | GATTTCCAAGTCTCCACCCCATTGACGTCAATGGGAGTTTGTTTTGGCACCAAAATCAACG | 61 | 93.4 | Nucleic acid fragments synthesized *in vitro* |
| (5'FAM) CMV-WT-R1 |  | CGTTGATTTTGGTGCCAAAACAAACTCCCATTGACGTCAATGGGGTGGAGACTTGGAAATC |  | 93.4 |  |
| ori-wt-F1 | U55762 | CTGCTGCTTGCAAACAAAAAAACCACCGCTACCAGCGGTGGTTTGTTTGCCGGATCAAGAG | 61 | 95.5 |  |
| ori-wt-R1 |  | CTCTTGATCCGGCAAACAAACCACCGCTGGTAGCGGTGGTTTTTTTGTTTGCAAGCAGCAG |  | 95.5 |  |
| HSV-wt-F1 | U55762 | GCTATGACGGCAATAAAAAGACAGAATAAAACGCACGGTGTTGGGTCGTTTGTTCATAAAC | 61 | 89.7 |  |
| HSV-wt-R1 |  | GTTTATGAACAAACGACCCAACACCGTGCGTTTTATTCTGTCTTTTTATTGCCGTCATAGC |  | 89.7 |  |
| (5'FAM) CMV-mut-F1 | U55762 | GATTTCCAAGTCTCCACCCCATTGACGTCAATGGGACAAACAATTGGCACCAAAATCAACG | 61 | 93.9 |  |
| (5'FAM) CMV-mut-R1 |  | CGTTGATTTTGGTGCCAATTGTTTGTCCCATTGACGTCAATGGGGTGGAGACTTGGAAATC |  | 93.9 |  |
| ori-mut-F1 | U55762 | CTGCTGCTTGCAAACAAAAAAACCACCGCTACCAGCGGTGCAAACAATGCCGGATCAAGAG | 61 | 95.9 |  |
| ori-mut-R1 |  | CTCTTGATCCGGCATTGTTTGCACCGCTGGTAGCGGTGGTTTTTTTGTTTGCAAGCAGCAG |  | 95.9 |  |
| HSV-mut-F1 | U55762 | GCTATGACGGCAATAAAAAGACAGAATAAAACGCACGGTGTTGGGTCCAAACAACATAAAC | 61 | 88.9 |  |
| HSV-mut-R1 |  | GTTTATGTTGTTTGGACCCAACACCGTGCGTTTTATTCTGTCTTTTTATTGCCGTCATAGC |  | 88.9 |  |

*Supplementary Table 2. Primers used in RT-PCR used in the study.*

| **Name** | **GenBank Accession numbers** | **Sequence** | **Amplicon Size (bp)** | **Tm (℃)** | **Function** |
| --- | --- | --- | --- | --- | --- |
| isg15-pro-F6 | NC_007116.7 | GCTGGCCAAACACACAGTAG | 650 | 59.41 | ChIP |
| isg15-pro-R6 |  | AGGCAGAACAAATCCAACAAAAA |  | 57.90 |  |
| foxo3b-pro-F3 | NC_007131.7 | CCTCTGTCAGTGCTGTGGAG | 178 | 60.04 | ChIP |
| foxo3b-pro-R3 |  | ACTGAGTGACTGGAGGAGGG |  | 60.25 |  |
| zgc26-pro-F3 | NC_007136.7 | TCCGGAAATCCCGGTTATGT | 96 | 58.79 | ChIP |
| zgc26-pro-R3 |  | TGCCAGGTGCAGCTACATAA |  | 59.96 |  |
| phlda3-pro-F4 | NC_007134.7 | CCAGAGCGCAGTTAAATAGGAC | 197 | 59.13 | ChIP |
| phlda3-pro-R4 |  | ACTTGGAAGTCCCGTAATCTGG |  | 59.76 |  |
| si11-pro-F3 | NC_007116.7 | AATCCGAGCGGTCTGGTTTC | 180 | 60.39 | ChIP |
| si11-pro-R3 |  | TGTTTTGACGTCCAGCCTCA |  | 59.82 |  |
| gapdh-qF | NM_213094.2 | CGCTGGCATCTCCCTCAA | 84 | 59.41 | qRT-PCR |
| gapdh-qR |  | TCAGCAACACGATGGCTGTAG |  | 60.67 |  |
| isg15-rt-F1 | NM_001204169.1 | GTCAAGAACGAGAAGGGCCA | 143 | 59.97 |  |
| isg15-rt-R1 |  | GACTCGAGCTGTCTGCCTTT |  | 60.04 |  |
| foxo3b-rt-F1 | NM_131085.1 | GAGCACCCCTGACAAGAGAC | 122 | 60.04 |  |
| foxo3b-rt-R1 |  | GCCGGATGGAGTTCTTCCAA |  | 60.04 |  |
| cdkn1a-rt-F1 | NM_001128420.1 | CTCCACACTTCCAGCTTCAGG | 189 | 60.61 |  |
| cdkn1a-rt-R1 |  | GGCCCATTACCGAGTGAACG |  | 61.09 |  |
| phlda3-rt-F1 | NM_001002455.1 | TTTGAGGATTTCTCAGCCCAG | 190 | 57.92 |  |
| phlda3-rt-R1 |  | CGTGGCGTTACACCAACAGA |  | 60.88 |  |
| zgc26-rt-F1 | NM_001172680.1 | AGACTCACTGAGGGTTGCTC | 168 | 59.03 |  |
| zgc26-rt-R1 |  | AAGAACCTGCCACACATCGC |  | 61.24 |  |
| si11-rt-F1 | NM_001109739.1 | TGACGCAGGATGCAGAATCAA | 108 | 60.34 |  |
| si11-rt-R1 |  | GAGGAAGAACCTTGCACCCA |  | 59.89 |  |
| hGAPDH-Q-F1 | NC_000012 | CACTAGGCGCTCACTGTTCTC | 101 | 60.73 |  |
| hGAPDH-Q-R1 |  | CCAATACGACCAAATCCGTTGAC |  | 60.18 |  |
| ISG15-RT-F1 | NC_000001.11 | GGTGGACAAATGCGACGAAC | 159 | 60.11 |  |
| ISG15-RT-R1 |  | TCGAAGGTCAGCCAGAACAG |  | 59.68 |  |
| ifnphi1-Q-F1 | NC_007114.7 | CTGACCTCAAAGAATGTGTGGC | 196 | 59.6 |  |
| ifnphi1-Q-R1 |  | CTTGCGTTGCTTGCGATGAT |  | 62.1 |  |
| ifnphi2-Q-F2 | NM_001111082.1 | CAACGACAGTTATCCGGAGG | 150 | 57.9 |  |
| ifnphi2-Q-R2 |  | TTCAGAGCATCGTCTCTTGC |  | 56.0 |  |
| ifnphi3-Q-F2 | NM_001111083.1 | GCATTGTTTGAGAACTTCGGT | 131 | 57.0 |  |
| ifnphi3-Q-R2 |  | AAATCCTGAGCCACTTCAGTC |  | 55.9 |  |
| ifnphi4-Q-F2 | NM_001161740.1 | CTCTGGTGGAAATCACTGCC | 125 | 57.6 |  |
| ifnphi4-Q-R2 |  | TTTGTAAGAGCCGCATGAGC |  | 59.0 |  |
| cgasa-Q-F1 | XM_680019.5 | TCCGAACGATCTAAAGCATCCA | 149 | 62.3 |  |
| cgasa-Q-R1 |  | TCATCTGGTTCACAAATTTTAAGGT |  | 59.5 |  |
| tnfa-Q-F2 | NM_212859.2 | GGGTGTTTGGTTTGTGAACG | 113 | 58.5 |  |
| tnfa-Q-R2 |  | CAATGTACAGATGTGTTGGCG |  | 57.2 |  |
| sting1-Q-F1 | NM_001278837.1 | TTGGCGAGAGAGAACGGAAG | 129 | 60.3 |  |
| sting1-Q-R1 |  | GATCACCTGTGTGTTCATCGTT |  | 57.1 |  |
| Kana-F1 | NZ_JAHLZF010000032.1 | CGATCAGGATGATCTGGACGAAG | 173 | 60.61 | Detection of plasmid residues by QPCR |
| Kana-R1 |  | GGCCACAGTCGATGAATCCAG |  | 61.08 |  |
| CMV-nick-F1 | NZ_JABAIK010000039.1 | GATAGCGGTTTGACTCACGGG | 112 | 61.07 |  |
| CMV-nick-R1 |  | GCGGAGTTGTTACGACATTTTGG |  | 60.91 |  |
| HSV-nick-F1 | NZ_JHAL01000002.1 | GCGCTATGACGGCAATAAAAAGACAG | 79 | 63.30 |  |
| HSV-nick-R1 |  | CAGAGTGCCAGCCCTGG |  | 59.68 |  |
| EGFP-RT-F1 | NZ_WTYD01000009.1 | CAACCACTACCTGAGCACCC | 112 | 60.32 |  |
| EGFP-RT-R1 |  | GTCCATGCCGAGAGTGATCC |  | 60.25 |  |

All sequences are synthesized in GenScript (China).
